# Community-Based Learning in a Time of Conflict

**DOI:** 10.1371/journal.pmed.0030115

**Published:** 2006-02-28

**Authors:** Bishnu Giri, P. Ravi Shankar

**Affiliations:** **1**Manipal College of Medical SciencesPokharaNepal

We read with interest the Editorial “Improving Health by Investing in Medical Education” [ 1]. The emphasis on problem-based, community-oriented, integrated teaching was of special interest. In Nepal, though community-based learning (CBL) has been carried out for many years, the majority of teaching still occurs in acute hospital settings. The ongoing conflict in Nepal has had an impact on all sectors, including medical education. CBL has been quite severely affected.

The Institute of Medicine (IOM), Kathmandu, the first medical college in Nepal, organizes community diagnosis programs (comprehensive assessments of the health status of a community in relation to its social, physical, and biological environment) [ 2].

The Manipal College of Medical Sciences (MCOMS), Pokhara, Nepal, admits mainly students from Nepal, India, and Sri Lanka for the undergraduate medical (MBBS) course. The revised curriculum of Kathmandu University [ 3], to which the college is affiliated, emphasises CBL. Community diagnosis, school health studies, family studies, participation in rural health camps, and health education are the various activities carried out. The Department of Pharmacology conducts exercises to acquaint students with the processes of the Community Drug Program (a system of community financing of drugs) and with the investigation of medicine use in peripheral health centres using drug-use indicators.

Around six years ago, when the insurgency was just taking root, students used to visit remote areas in Kaski (the district which includes the city of Pokhara) and neighbouring districts as a part of their community diagnosis studies. They stayed there for a few days, interacting closely with local communities. Community field trips were, however, gradually curtailed with the rise of the insurgency, and were finally limited to the Pokhara Valley. The college authorities were apprehensive about the possible harm to students and faculty members. Other colleges have also curtailed their CBL because of the spread of the insurgency. A friend from IOM wrote recently saying, “The prevailing situation is affecting the site selection for community studies. The studies used to be conducted at sites outside the Kathmandu Valley, but these days the sites are preferentially chosen inside the valley” (S. Gurung, personal communication).

In private medical colleges, students mainly come from economically well-off families and have little idea of poverty and deprivation. Some of my friends were surprised to see a family living in a small hut on a hillside in a village just outside Pokhara. Young women often give birth without trained medical assistance in rural areas. Women carry heavy loads of firewood and fodder for their cattle along steep mountain trails well into late pregnancy. Empathy and sympathy for the less fortunate may be qualities lacking in doctors who are not exposed to rural life. Their impression of the community's health status may be lopsided. In their future practice, students may not consider the patient's economic status while prescribing treatment.

The deleterious effects of conflict on health status of a community are an established fact. Urban students having little experience in rural Nepal may find it difficult to adjust if posted to a rural health centre after graduation. The present curtailment of CBL in rural areas may lead to production of less competent manpower and ineffective policymaking in the not too distant future. We sincerely hope that, with the hope of peace being reestablished, CBL in Nepal will be strengthened and expanded.
